# Optimization of In Vitro Germination, Viability Tests and Storage of Daylily (*Hemerocallis* spp.) Pollen

**DOI:** 10.3390/plants14121854

**Published:** 2025-06-16

**Authors:** Wei Li, Chongcheng Yang, Jiyuan Li, Lixin Huang, Jinsong Guo, Feng Feng

**Affiliations:** College of Coastal Agricultural Sciences, Guangdong Ocean University, Zhanjiang 524088, China; 17820335211@163.com (W.L.); 15014960216@stu.gdou.edu.cn (C.Y.); livia7118@163.com (J.L.); 202111321109@stu.gdou.edu.cn (L.H.); 17506063695@163.com (J.G.)

**Keywords:** Daylily (*Hemerocallis* spp.), optimal medium, pollen viability, temperature, storage conditions

## Abstract

Daylily (*Hemerocallis* spp.) are perennial herbaceous flowers with high ornamental and medicinal value. Currently, the breeding of new daylily cultivars was mainly achieved through hybrid breeding, but issues such as self-incompatibility, hybridization barriers, and asynchronous reproductive phenology severely hinder the breeding process. Understanding pollen viability was essential for daylily breeding and cultivar improvement. In this study, we systematically investigated the effects of pollen viability determination methods, collection time, medium combinations, culture temperature and storage conditions on the pollen germination characteristics of daylily, using five daylily cultivars introduced in the Zhanjiang region of China as materials. Comparing the Iodine-potassium iodide (I_2_-KI) staining and Acetocarmine staining, the results of 2,3,5-Triphenyltetrazolium Chloride (TTC) staining showed a significant positive correlation (*p* < 0.05) with the in vitro germination rate, which is suitable for the rapid detection of daylily pollen vigor. The daylily variation of pollen vigor was significant in different cultivars, and most cultivars had the highest vigor at 9:00–12:00 a.m., which was suitable for artificial pollination. The in vitro germination experiment showed that sucrose concentration was the key factor for daylily pollen germination and pollen tube growth, and the optimal medium for pollen in vitro germination was 50 g/L^−1^ sucrose + 0.1 g/L^−1^ H_3_BO_3_ + 0.06 g/L^−1^ KNO_3_ + 0.2 g/L^−1^ Ca(NO_3_)_2_. The temperature experiment showed that the optimum temperature for pollen germination was 24.1–26.7 °C, and the optimum range for pollen tube growth was 24.1–25.7 °C, and the high temperature significantly inhibited the elongation rate of pollen tube. Storage experiments showed that low temperature (−40 °C) combined with drying treatment could significantly prolong pollen life, and the “Water Dragon” variety still maintained 41.29% vigor after 60 days of dry storage. This study provides theoretical basis and technical support for the introduction and domestication of daylily in South China, hybridization and garden application.

## 1. Introduction

Daylily (*Hemerocallis* spp.) are perennial herbs in the genus *Hemerocallis* of the family Asphodelaceae, which has high economic value [1]. Daylilies grow at altitudes of 300–2500 m in forests, grasslands, along streams, or under shrubs. Wild species are found south of the Qinling Mountains in China, and cultivated species are found throughout China [2]. Because of its ornamental, edible and medicinal values, daylily has been loved and utilized in China throughout history, and has been called the ‘Chinese Mother’s Flower’ [3]. Daylily has large and colorful flowers, is characterized by loose cultivation and management, barrenness tolerance, and strong adaptability, and is widely distributed around the world. It is the preferred flower material for domestic and international landscape gardening, and has a very broad application prospect [4].

Selection of new daylily cultivars is mainly realized through crossbreeding techniques, and the level of parental fertility is a decisive factor affecting the fruiting rate after cross-pollination [5]. Daylily flowering occurs primarily during the high-temperature seasons of summer and autumn, and prolonged high temperatures are widely recognized as the key factor causing pollen and ovule degeneration and a sharp decline in vitality [6]. This limiting factor, along with self-incompatibility, reproductive phenological asynchrony, and obstacles to distant hybridization, severely constrains efficient breeding progress [7,8]. Therefore, there is an urgent need to develop effective strategies to overcome the negative impact of high temperatures on pollen viability, such as optimizing pollen germination conditions in vitro, establishing reliable viability testing methods, and developing storage technologies to extend the effective storage period of pollen [9]. Although there have been related reports on pollen viability and pollen storage in daylily. Wang et al. (2009) investigated the effects of sucrose, boric acid, and calcium ions on the pollen viability of gold-dollar daylily (Hemerocallis ‘Stella de Oro’) [10]; Zhao et al. (2017) assessed pollen viability of six daylily cultivars by using TTC staining and optimize their germination medium [11]; Wang et al. (2019) systematically resolved the influence law of temperature gradient (−75 °C to 25 °C) on pollen viability [12]; And Zhou et al. (2022) established a hybrid parental screening system through pollen-stigma interactions analysis [3]. However, systematic research on the pollen fertility of daylilies after introduction into specific ecological zones, such as the high-temperature, high-humidity region of southern China, remains lacking.

In this study, four *Hemerocallis hybridus* cultivars with good trait performance introduced from Zhanjiang, China, and *Hemerocallis fulva*, a widely cultivated variety locally, were used as experimental materials to study the effects of different pollen viability assays, different time of collection, different storage conditions, different media combinations and different incubation temperatures on pollen viability and germination rate, in order to find out the best pollen staining method, pollen collection time, storage conditions and the best medium combination and temperature conditions for pollen germination, and to quantify the effect of temperature on the pollen germination of daylily, we will provide a scientific theoretical and practical basis for the introduction and domestication of daylily in Zhanjiang and even in South China, as well as for the breeding of hybrid breeding and the landscape application.

## 2. Results and Analysis

### 2.1. Optimization of Culture Medium for Pollen Germination In Vitro

#### 2.1.1. Effect of Different Media on Pollen Germination

As can be seen from Table 1, the differences in pollen germination rates of the five different daylily cultivars in different media were more obvious, and the pollen germination rates in No. 1 medium were all significantly higher than those of the other treatments. Under optimal culture conditions, pollen germination rates were as follows: *H*. ‘Water Dragon’ > *H*. ‘Prairie Belle’ > *H*. ‘Kimberly’ > *H*. ‘Golden Coffee Ruffles’ > *Hemerocallis fulva*, with germination rates of 62.65%, 48.91%, 42.78%, 36.43%, and 28.82%, respectively. The pollen of *H*. ‘Prairie Belle’ exhibited the lowest germination rate on Medium No. 7, at 13.23%. *H*. ‘Prairie Belle’ had the lowest germination rate on medium No. 5, at 16.4%. The three cultivars *H*. ‘Golden Coffee Ruffles’, *Hemerocallis fulva*, and *H*. ‘Kimberly’ had the lowest germination rates on medium No. 9, at 9.38%, 5.88%, and 13.92%, respectively. Analysis of the range and multiple variance of the orthogonal test results (Table 2 and Table 3) indicated that sucrose had the most significant effect on pollen germination, while Ca(NO_3_)_2_ had no significant effect on pollen germination. The effects of H_3_BO_3_ and KNO_3_ on pollen germination varied according to the cultivars, in which germination rate decreased with the increase in the concentration level of H_3_BO_3_.

#### 2.1.2. Effects of Different Media on Pollen Tube Growth

**Figure 1:**
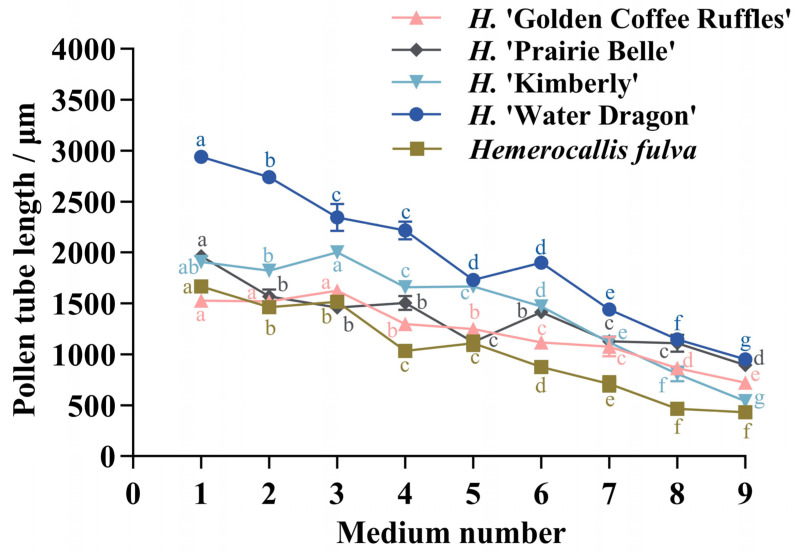
Pollen tube length of five different cultivars of daylily on different media. Different lowercase letters indicate significant differences at the 0.05 level.

**Figure 2:**
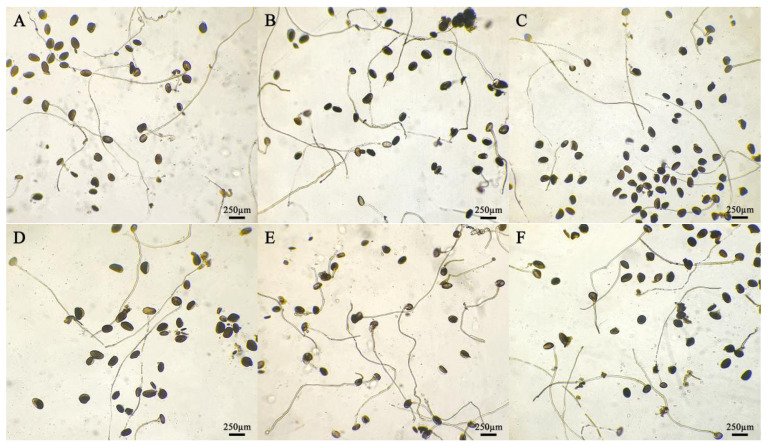

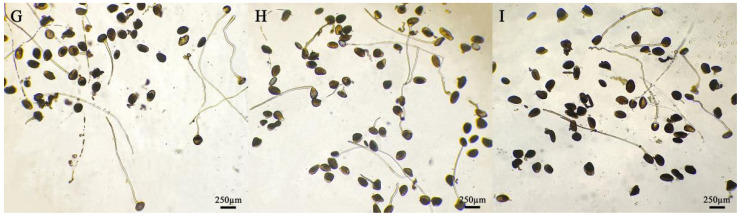
Germination of daylily pollen in different media. Medium: (**A**) No. 1; (**B**) No. 2; (**C**) No. 3; (**D**) No. 4; (**E**) No. 5; (**F**) No. 6; (**G**) No. 7; (**H**) No. 8; (**I**) No. 9. Pollen after 4 h of cultivation.

The pollen tube lengths of the five daylily cultivars in different medium treatments were significantly different (Figure 1). The pollen tube lengths of the five daylily cultivars were lower than the other eight treatments on medium No. 9, and the pollen tube lengths of *H.* ‘Water Dragon’, *Hemerocallis fulva* and *H.* ‘Prairie Belle’ was higher on medium No. 1 than the other treatments, which were 2940.75 ± 61.66 μm, 1665.75 ± 37.79 μm and 1965 ± 51.71 μm, respectively, *H.* ‘Water Dragon’ had pollen tube lengths greater than the other four cultivars on nine different medium treatments; The pollen tube length of *H.* ‘Golden Coffee Ruffles’ and *H.* ‘Kimberly’ was slightly higher than that of other treatments on culture medium No. 3, which were 1624.5 ± 28.31 μm and 2001 ± 51.48 μm respectively. The germination situation is shown in Figure 2. The ANOVA showed that sucrose concentration had a significant effect on the pollen tube growth of the five daylily cultivars. A correlation analysis was conducted between different concentrations of sucrose and the pollen tube length of 5 daylily cultivars. Sucrose was extremely significantly negatively correlated with *H.* ‘Golden Coffee Ruffles’ and *H.* ‘Prairie Belle’ (*p* < 0.01) and significantly negatively correlated with *H.* ‘Water Dragon’ and *Hemerocallis fulva* (*p* < 0.05). When the concentration of H_3_BO_3_ was 0.1 g·L^−1^, the length of pollen tube was slightly longer than the two levels of K2 and K3, which indicated that the high concentration of H_3_BO_3_ would also inhibit the growth of pollen tube. The effect of different concentration levels of KNO_3_ on the growth of pollen tube varied according to the cultivars, and the effect of adding Ca(NO_3_)_2_ on the growth of pollen tube was not significant (Table 4).

### 2.2. Effects of Different Culture Temperatures on Daylily Pollen Germination and Pollen Tube Growth

**Figure 3:**
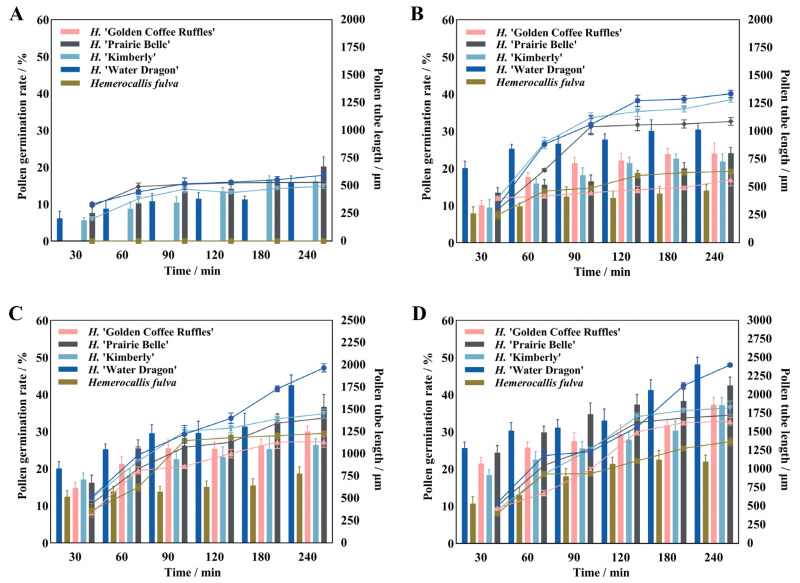

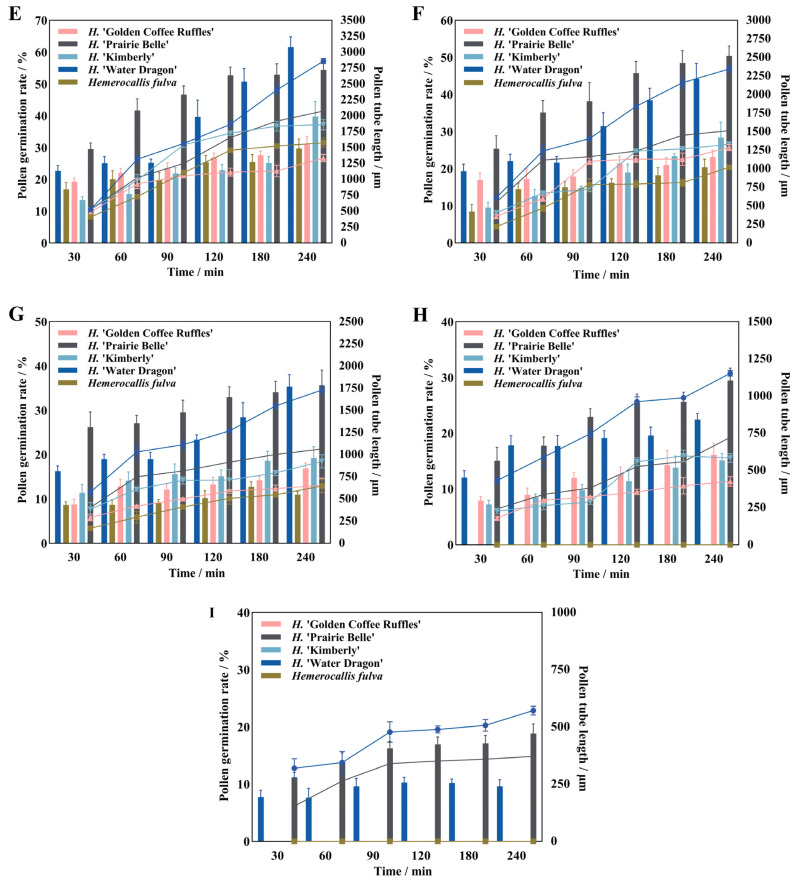
Pollen germination rate and pollen tube length of daylily at different temperatures. Note: (**A**) 14 °C; (**B**) 17 °C; (**C**) 20 °C; (**D**) 23 °C; (**E**) 26 °C; (**F**) 29 °C; (**G**) 32 °C; (**H)** 35 °C; (**I**) 38 °C.

Different culture temperatures have certain effects on the pollen germination rate, pollen tube growth and elongation rate of daylily. There were significant differences in pollen germination rate and pollen tube length between different cultivars at different culture temperatures (Figure 3). Both *H*. ‘Golden Coffee Ruffles’ and *Hemerocallis fulva* had no pollen germination at 14 °C, and then the pollen germination rate and pollen tube length increased significantly with the increase of temperature, and the former had the highest pollen germination rate and pollen tube length (37.37%, 1644.75 μm) when the temperature reached 23 °C. The latter reached the highest (29.72%, 1575 μm) at 26 °C, and there was no pollen germination at 35 °C. *H*. ‘Water Dragon’, *H*. ‘Kimberly’, and *H*. ‘Prairie Belle’ all exhibited pollen germination at 14 °C, reached their peak at 26 °C, and then rapidly declined as temperature increased. Among these, the pollen of *H*. ‘Prairie Belle’ and *H*. ‘Water Dragon’ remained viable at 38 °C, with viability rates of 18.86% and 9.6%, respectively. Pollen tube lengths were 371.25 ± 40.94 μm and 571.5 ± 19.14 μm, respectively. The total rate of pollen tube elongation of *H*. ‘Water Dragon’ was greater than that of the other four cultivars, reaching a maximum of 11.92 μm/min at 26 °C, with the fastest elongation of 26.38 μm/min in 30–60 min. *H*. ‘Prairie Belle’, *H*. ‘Kimberly’ and *Hemerocallis fulva* all reached their maximum total pollen tube elongation rates at 26 °C, which were 8.64 μm/min, 7.75 μm/min and 6.56 μm/min, while *H*. ‘Golden Coffee Ruffles’ reached its maximum value of 6.85 μm/min at 23 °C.

**Figure 4:**
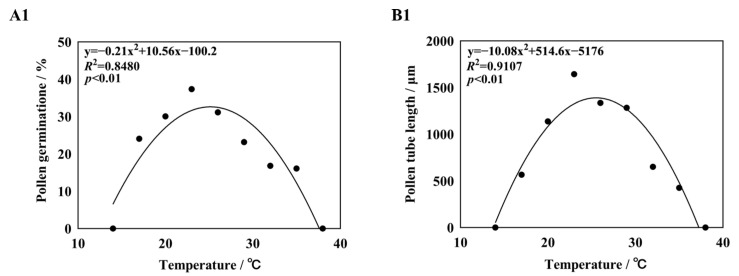

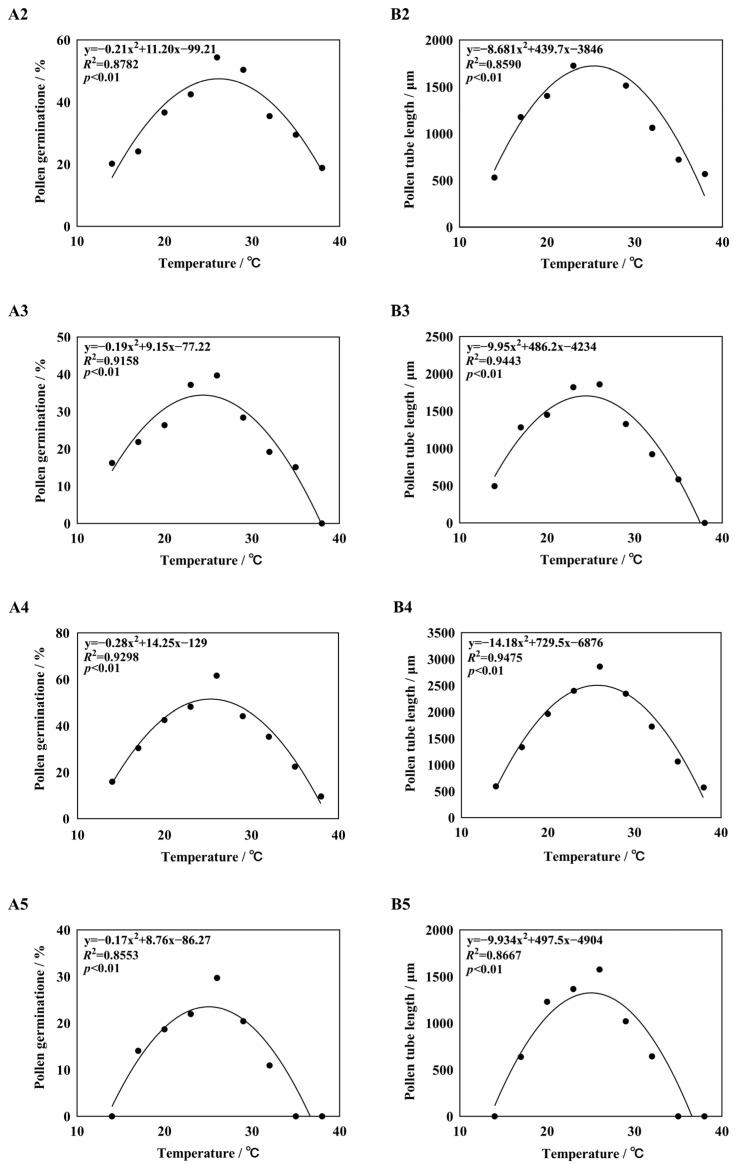
Nonlinear regression obtain for pollen germination rate and pollen tube length in response to temperature. Note: (**A1**,**B1**) *H.* ‘Golden Coffee Ruffles’; (**A2**,**B2**) *H.* ‘Prairie Belle’; (**A3**,**B3**) *H.* ‘Kimberly’; (**A4**,**B4**) *H.* ‘Water Dragon’; (**A5**,**B5**): *Hemerocallis fulva*.

**Figure 5:**
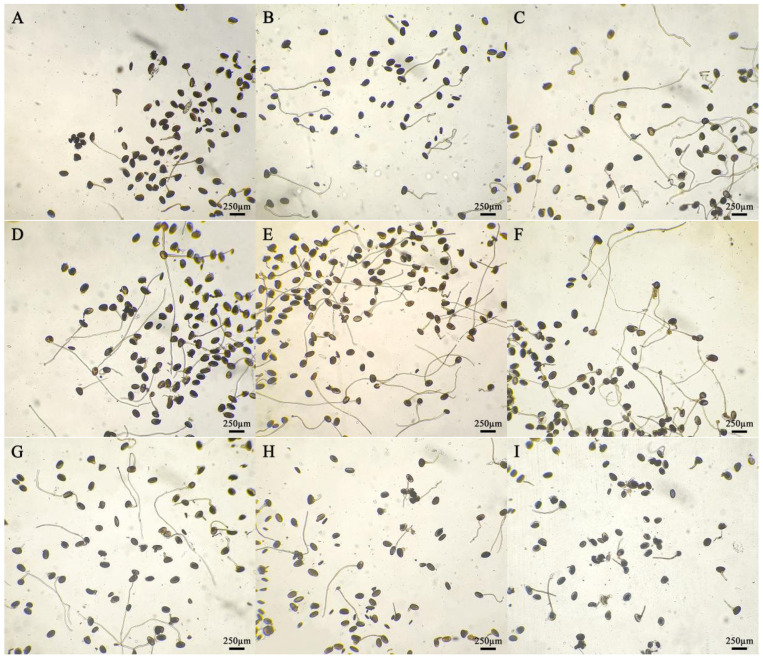
Germination of daylilies at different temperatures. Note: (**A**) 14 °C; (**B**) 17 °C; (**C**) 20 °C; (**D**) 23 °C; (**E**) 26 °C; (**F**) 29 °C; (**G**) 32 °C; (**H**) 35 °C; (**I**) 38 °C.

Curves were fitted to the germination rates and pollen tube lengths of five different daylily cultivars (Figure 4). It was found that the quadratic term equation could fit the germination rate and pollen tube length better, and the *R*^2^ values were all greater than 0.80, *p* < 0.01, reaching a significant level. The quadratic term equation was used to predict the basic temperature of pollen germination and pollen tube growth, with Topt being the optimal temperature for pollen germination or pollen tube growth, Tmin being the lowest temperature at which pollen started to germinate or pollen tube growth, and Tmax being the highest temperature. The temperature range of pollen germination of five different daylily cultivars was 10.91–42.12 °C, the optimum temperature was 24.08–26.67 °C, and the germination rate in this temperature range was 26.58–52.31%. The optimum temperature range for pollen tube growth was 24.07–25.66 °C, and the length of pollen tubes in this temperature range was 1324.78–2506.41 μm (Supplementary Table S1). The germination is shown in Figure 5.

### 2.3. Screening of Suitable Pollen Viability Assay Methods

**Figure 6:**
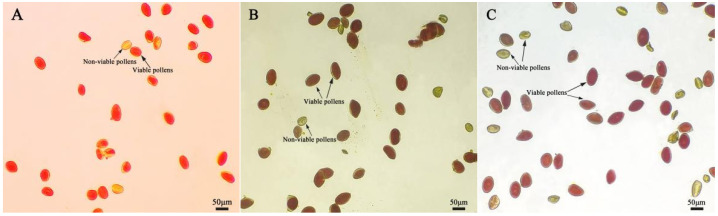
Detection of pollen viability by different staining methods. (**A**) Acetocarmine staining method; (**B**) I_2_-KI staining method; (**C**) TTC staining method. Red, dark red, and reddish brown pollen: Viable pollens; yellow and yellowish brown: Non-viable pollens.

**Figure 7:**
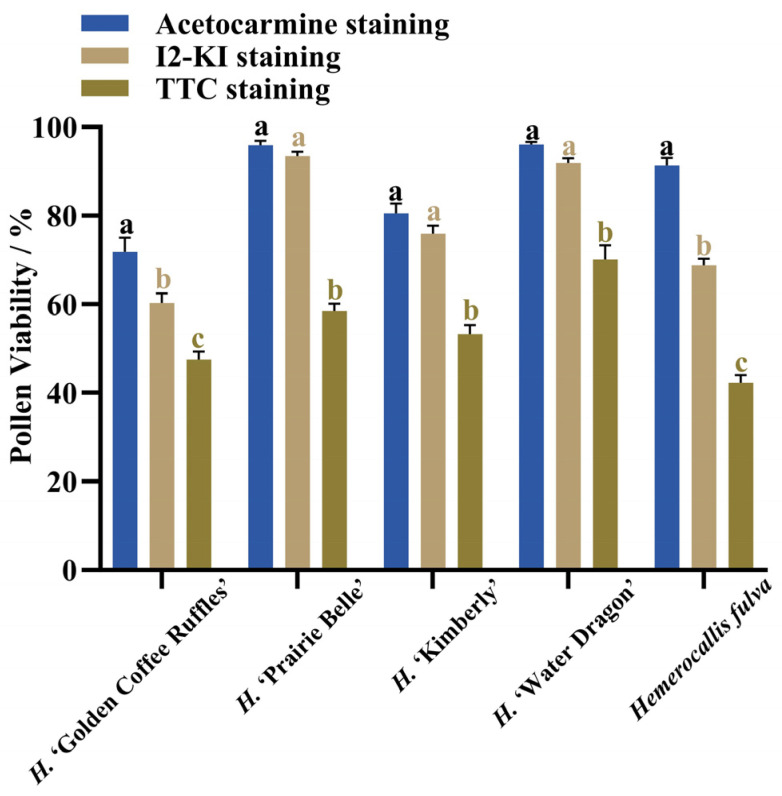
Pollen viability as determined by different staining methods. Different lowercase letters indicate significant differences at the 0.05 level.

**Figure 8:**
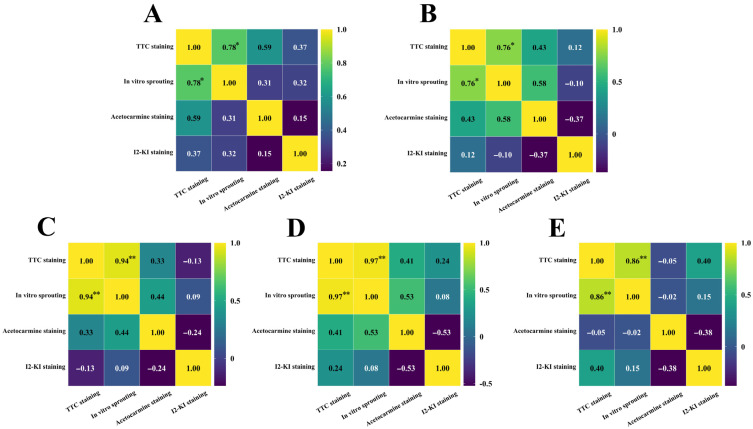
Correlation analysis between different staining methods and in vitro germination rates. (**A**) *H*. ‘Golden Coffee Ruffles’; (**B**) *H*. ‘Prairie Belle’; (**C**) *H*. ‘Kimberly’; (**D**) *H*. ‘Water Dragon’; (**E**) *Hemerocallis fulva*. "*" indicates a significant difference (*p* < 0.05), "**" indicates very significant difference (*p* < 0.01).

**Figure 9:**
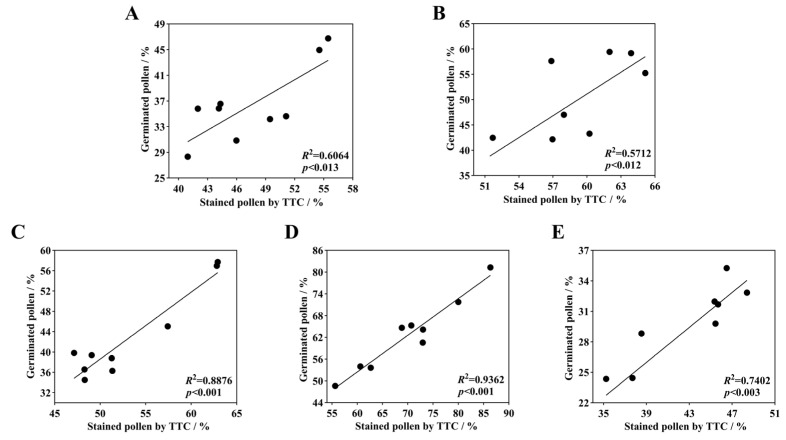
Linear regression of daylily isolated germinated pollen with TTC-stained pollen. (**A**) *H.* ‘Golden Coffee Ruffles’; (**B**) *H.* ‘Prairie Belle’; (**C**) *H.* ‘Kimberly’; (**D**) *H.* ‘Water Dragon’; (**E**) *Hemerocallis fulva*.

After staining daylily pollen by using three commonly used pollen staining methods, it was found that Acetocarmine, I2-KI and TTC were able to differentiate between viable and non-viable pollen (Figure 6A–C). After staining with acetocarmine, pollen grains were stained red with very few yellow pollen grains, which were regarded as non-viable pollen grains (Figure 6A). After I_2_-KI staining, pollen grains were stained reddish brown or red, with very few yellowish brown pollen grains and shriveled morphology, which were regarded as non-viable pollen grains (Figure 6B). After TTC staining, viable pollen grains were stained dark red or rosy red, less viable pollen grains were stained light red, and non-viable pollen grains were stained yellow-brown or not stained (Figure 6C), in which the values of viability of pollen stained with acetocarmine and I_2_-KI were large (Figure 7). The correlation matrix analysis of the three pollen staining viability and the isolated germination test revealed that the TTC staining method was significantly or highly significantly positively correlated with the pollen isolated germination method, and that there was no significant difference between the acetocarmine and I_2_-KI and the isolated germination method (Figure 8). Further linear regression analysis of the TTC staining method and the isolated germination method revealed that the *p* values were less than 0.05 or less than 0.001, and it can be considered that there was a linear correlation between the two (Figure 9).

### 2.4. Effects of Different Collection Times on Daylily Pollen Viability

**Figure 10:**
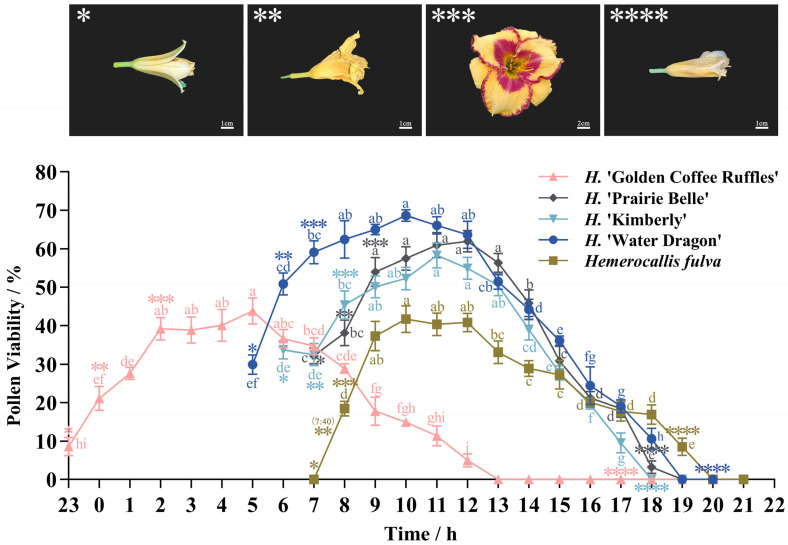
The Pollen viability of five daylily cultivars at different times of collection. Daylily blooming process. *: Bud break; **: Petal spread; ***: Full scale; ****: Flowers wither. Different lowercase letters indicate significant differences at the 0.05 level.

Daylily pollen viability varied greatly among different cultivars at different times (Figure 10). Among the five daylily cultivars measured, the overall trend of pollen vitality first increased and then decreased. The *H*. ‘Golden Coffee Ruffles’ has a distinct difference in blooming time compared to other cultivars, with flower buds opening at night; The *H*. ‘Golden Coffee Ruffles’ reaches its maximum pollen viability at 5:00 a.m. (43.82%), after which it declines significantly and loses viability after 8 h; The *H*. ‘Water Dragon’ flower buds begin to open at 5:00 a.m., with pollen viability peaking at 10:00 a.m. (68.65%); The *H*. ‘Water Dragon’ maintains pollen viability for 14 h after flower bud opening, with pollen viability peaks higher than other cultivars; The *H*. ‘Prairie Belle’ and *H*. ‘Kimberly’ maintain pollen viability for 12 h after bud opening, with pollen viability at bud opening higher than the other three cultivars, at 32.15% and 33.69%, respectively; The *H*. ‘Prairie Belle’ and *H*. ‘Kimberly’ reach peak pollen viability at 10:00 a.m. and 11:00 a.m., at 61.98% and 58.31%, respectively, and pollen viability began to decline significantly after 12:00 a.m.; The buds of the *Hemerocallis fulva* began to open at 7:00 a.m., at which time the pollen was not detected as viable. At 8:00 a.m., viability began to rise rapidly, reaching a maximum of 41.71% at 10:00 a.m. After 12:00 p.m., viability began to decline slowly. The *H*. ‘Golden Coffee Ruffles’ showed high pollen viability throughout the 2:00–5:00 time period, while the pollen viability of the remaining four daylily cultivars remained at high levels between 9:00 and 12:00. The pollen viability of the five daylily cultivars during the four time periods with the highest viability was not significantly different (*p* < 0.05), indicating that the five daylily cultivars can maintain their highest viability for four hours.

### 2.5. Effect of Storage Temperature and Storage Time on Pollen Viability

**Figure 11:**
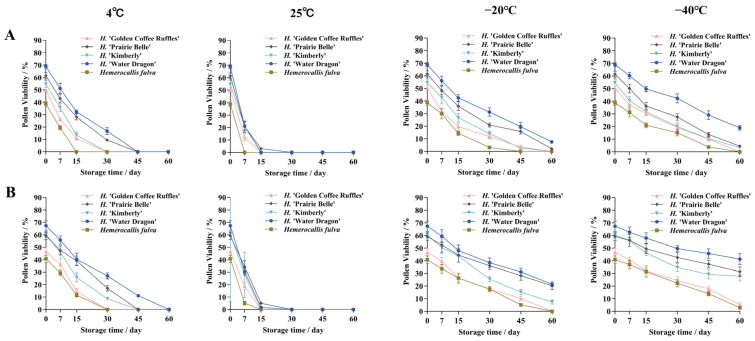
Pollen viability of five daylily cultivars under different storage conditions. (**A**) Undried pollen viability; (**B**) Pollen viability of dried treatments.

Different storage conditions and storage time had significant effects on daylily pollen vigor (Figure 11). Analysis of variance (ANOVA) showed that the effect of storage time on pollen vigor was more significant than that of storage conditions (Supplementary Table S2). Under different storage conditions, pollen viability showed a decreasing trend with the change of storage time, the lower the temperature, the slower the decreasing trend, the pollen viability of the dried pollen was higher than that of the non-dried pollen, and the pollen storage characteristics were different among different cultivars.

#### 2.5.1. Effects of 25 °C and 4 °C Storage Conditions on Pollen Viability

The pollen viability under 25 °C and 4 °C storage conditions decreased rapidly with the prolongation of storage time, among which the pollen viability decreased the fastest under 25 °C. Under 25 °C storage conditions, the pollen viability of five daylily cultivars decreased rapidly from 0 to 7 d, and completely lost its viability at 30 d of storage. The pollen of *H*. ‘Water Dragon’ was able to be stored for a longer period of time under 4 °C storage conditions, and the pollen was still viable at 45 d of storage, which was 11.07% under the drying treatment; the pollen of *H*. ‘Prairie Belle’ and *H*. ‘Kimberly’ had pollen viability of 17.06% and 8.67%, respectively, at 30 d of dry storage; *Hemerocallis fulva* and *H*. ‘Golden Coffee Ruffles’ had declining pollen viability The pollen viability of *Hemerocallis fulva* and *H*. ‘Golden Coffee Ruffles’ had the fastest decreasing trend, and the pollen completely lost its viability at 30 d of storage.

#### 2.5.2. Effects of −20 °C and −40 °C Storage Conditions on Pollen Viability

Pollen viability under −20 °C and −40 °C storage conditions decreased more gently with the extension of storage time, in which the slowest trend of decrease in pollen viability was observed under −40 °C, which was significantly higher than other storage conditions. Under −40 °C storage conditions, the pollen viability of *H*. ‘Water Dragon’, *H*. ‘Prairie Belle’ and *H*. ‘Kimberly’ decreased in a slower trend, and the pollen viability under drying treatment decreased in a slower trend. The trend was slower, and the pollen viability was 41.29%, 31.31 and 27.78 at 60 d of storage under drying treatment, while *Hemerocallis fulva* and *H*. ‘Golden Coffee Ruffles’ decreased to 2.83% pollen viability at 60 d of storage under drying treatment. Pollen viability decreased to 2.83% and 5.15%, and the pollen without drying treatment completely lost its viability. The pollen viability of *H*. ‘Water Dragon’ and *H*. ‘Prairie Belle’ was significantly higher than that of the other three cultivars when stored at −20 °C for 60 d under drying treatment, the pollen viability was 21.41% and 20.20%, respectively. 20.20% at 60 d of storage under drying treatment, and 7.56% and 2.06% without drying treatment, respectively; *H*. ‘Golden Coffee Ruffles’ and *Hemerocallis fulva* lost pollen viability completely at 60 d of storage.

## 3. Discussion

### 3.1. Pollen In Vitro Germination Culture

It has been shown that the germination rate of plant pollen affects the fruiting rate of heterogamous pollination [13]. In the process of introduction and production, daylily’s flower failure is a common problem, and understanding daylily’s pollen isolation and germination characteristics is important to improve the efficiency of crossbreeding. The composition of pollen germination media varies among plants and species, and different combinations of components affect in vitro germination [14]. Sucrose, H_3_BO_3_, Ca^2+^, Mg^2+^ and K^+^ are the most commonly used additives in in vitro pollen germination assays [15,16]. Among them, sucrose not only provides the necessary energy for pollen germination and pollen tube growth, but also regulates the osmotic pressure of the medium and prevents pollen from rupturing due to excessive water uptake [17]. The results showed that the addition of sucrose, H_3_BO_3_, Ca_2_ + and K+ all had an effect on daylily pollen germination, and the optimal combination was 50 g/L^−1^ sucrose + 0.1 g/L^−1^ H_3_BO_3_ + 0.06 g/L^−1^KNO_3_ + 0.2 g/L^−1^ Ca(NO_3_)_2_, and all five daylily cultivars obtained the highest germination rate on this formulation. Our results showed that sucrose was the main factor affecting pollen germination and pollen tube elongation, and excessive sucrose concentration would inhibit daylily pollen germination and pollen tube elongation. Meanwhile, high concentration of H_3_BO_3_ (0.3 g·L^−1^) also inhibited pollen germination and pollen tube elongation, which was consistent with the results of studies on pollen germination of *Exochorda racemosa* [16], *Paeonia ludlowii* [18], and *Phoenix dactylifera* [19].

### 3.2. Effect of Different Incubation Temperatures on Pollination

Plants are most sensitive to temperature during the reproductive stage of development, and high or low temperatures can directly affect fertilization and fruit set [20,21]. During flowering, pollen is susceptible to temperature extremes, and high or low temperatures can lead to a decrease in pollen germination. Studies have shown that the optimum temperature for pollen germination in most plants is 20–28 °C [6,16,22], and a small number of plants require lower temperatures for pollen germination [23]. Currently, there are no relevant studies on the effect of temperature on daylily plants. Therefore, the main purpose of this study was to investigate the effects of different temperatures on pollen germination rate and pollen tube growth of introduced daylily cultivars by simulating the climatic conditions (14–38 °C) in Zhanjiang area. The results showed that there were significant differences in pollen germination and pollen tube length among different cultivars at different temperatures, indicating differences in pollen tolerance among different cultivars and among different cultivars of the same cultivars [24,25,26]. Among them, *H*. ‘Water Dragon’ (Tmin = 11.78, Tmax = 39.11) and *H*. ‘Prairie Belle’ (Tmin = 11.21, Tmax = 42.12) exhibited good germination rates (15.96–20.21% and 9.60–18.86%, respectively) under low-temperature (14 °C) and high-temperature (38 °C) conditions. At optimal temperatures, the average pollen tube lengths for these two varieties were 2506.41 μm and 1720.60 μm, respectively, significantly higher than those of the other three varieties. Therefore, these two varieties can be continuously introduced as heat-tolerant varieties to enhance their pollen’s survival capacity under extreme environmental conditions. The average germination rate of the local variety Daylily (Tmin = 13.26, Tmax = 38.26) was only 26.58%, and the pollen completely lost its ability to germinate at a high temperature of 35 °C, and the average length of pollen tube was 1324.78 μm, which was significantly lower than the other four cultivars. *H*. ‘Golden Coffee Ruffles’ (Tmin = 12.69, Tmax = 37.59) and *H*. ‘Kimberly’ (Tmin = 10.91, Tmax = 37.25) had mean germination rates were not significantly different, 32.55% and 32.94%, respectively, and the latter had a longer mean pollen tube length of 1705.46 μm. In summary, the five different cultivars of daylily had significant temperature optima for pollen germination and pollen tube growth (24.08–26.67 °C, 24.07–25.66 °C), and above and below this temperature optimum, both pollen germination rate and pollen tube length both decreased, and this response can be well described by a quadratic linear regression model. Maximum pollen germination rate and pollen tube length as well as Tmin, Tmax and Topt among different cultivars are the most important parameters describing the low and high temperature tolerance of plant cultivars, revealing the adaptation of genotypes to site-specific temperature conditions [21,27,28]. Therefore, the adaptation of cultivars to temperature should be taken into account when making introductions of daylily plants in a particular region.

### 3.3. Effect of Staining Method and Collection Time on Pollen Viability

Pollen staining methods have the advantages of being easy to use and time-consuming in pollen viability assays [29,30]. However, different staining reagents have different mechanisms of action and are susceptible to interference by the inherent properties of pollen, leading to differences in the results [31]. In this paper, daylily pollen viability was determined by using three stains, and the pollen viability values stained by acetocarmine and I_2_-KI were high compared with the results of preexisting pollen in vitro germination, while the differences between TTC staining and the results of pollen in vitro germination were small and there was a linear correlation between the two, so that the TTC staining method is the most suitable for the rapid detection of daylily pollen viability. The results of this study are similar to those of other pollen viability studies [5,32,33].

Determination of the pollen collection cycle is also crucial in order to ensure the success rate of artificial pollination [34]. The single flowering period of daylilies does not exceed 24 h, therefore, in order to identify the time of day when pollen viability is the highest, with a view to providing efficient pollen for artificial pollination. In this study, the flowering time of different cultivars of daylilies was observed by sampling at the time of initial unfolding of the flower buds, and then at hourly intervals until the flowers wilted. The results showed that the pollen viability of different cultivars varied significantly across time, and the overall trend of pollen viability was first increasing and then decreasing, consistent with previous studies [11,34]. *H*. ‘Golden Coffee Ruffles’ and *H*. ‘Water Dragon’ both belonged to night-flowering cultivars, with pollen vigor of the former peaking at 5:00 (43.82%) and then declining slowly; the latter In the former, pollen viability peaked at 5:00 (43.82%) and then declined slowly, while in the latter, high viability was detected at the first opening of the buds at 5:00 (29.9%) and peaked at 10:00 (68.65%). Pollen viability of *H*. ‘Kimberly’, *H*. ‘Prairie Belle’, and *H*. ‘Daylily’ peaked at 10:00–12:00, at 58.31%, 61.98% and 41.71%. Except for *H*. ‘Golden Coffee Ruffles’, the other four cultivars maintained high vigor from 9:00–12:00, which can be the best time for pollen collection.

### 3.4. Pollen Storage Conditions

Crossbreeding is one of the most commonly used means of breeding new cultivars of plants [35,36], and different flowering times between different cultivars of daylilies often encounter problems of incompatible flowering, cross-seasonal or long-distance hybridization during the crossbreeding process, which requires the collection and storage of pollen [10,12,37]. The results of this study showed that the pollen storage vigor of different cultivars of daylilies differed significantly depending on the variety, storage conditions and storage time. The results of the present study are in agreement with the results of other authors [38,39,40,41,42]. Compared to fresh pollen, different cultivars of daylily showed a decrease in pollen viability after preserving pollen for 0–60 days under eight storage conditions. The pollen viability of pollen stored with drying treatment was all greater than that of pollen stored without drying treatment, so pollen longevity can be extended when pollen moisture content is reduced and stored at low temperatures [38,43]. The results showed that the pollen viability of five daylily cultivars was 2.83–41.29% after 60 days of storage at −40 °C, while the viability of pollen without drying treatment was still 3.48–19.03%. This indicated that the life span of daylily pollen could be more than 60 days under −40 °C storage condition, which was suitable for long-term preservation of pollen. Storage of pollen at −20 °C is also a simple way to maintain pollen viability. Compared with storage at 4 °C, the pollen viability of five daylily cultivars after 45 days of dry storage at −20 °C ranged from 5.07–31.11%, which was significantly higher than that of storage at 4 °C, while the pollen viability of pollen after 15 days of storage at 25 °C declined rapidly, and was only 1.73–5.09%. Pollen viability decreased more rapidly in pollen stored at room temperature 25 °C compared to pollen stored at lower temperatures [39,44]. Our results are also consistent with those of Bai et al. (2023), Wang et al. (2019), and Jung et al. (2003), who obtained higher pollen viability under −20 °C, −75 °C, −80 °C and storage, respectively, but lower at 4 and 25 degree [12,45,46].

## 4. Materials and Methods

### 4.1. Plant Material and Pollen Collection

**Figure 12:**
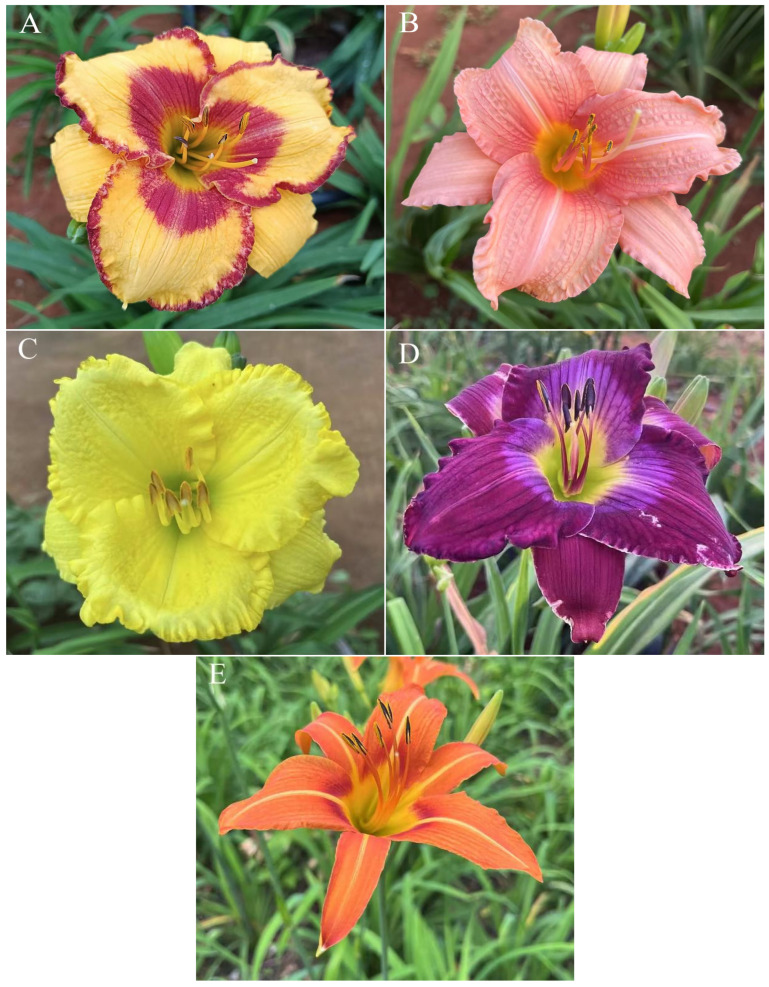
5 Daylily cultivars. (**A**) *H*. ‘Golden Coffee Ruffles’; (**B**) *H*. ‘Prairie Belle’; (**C**) *H*. ‘Kimberly’; (**D**) *H*. ‘Water Dragon’; (**E**) *Hemerocallis fulva*.

Five cultivars of daylily planted in the daylily resource nursery in the Forest and Fruit Building of Guangdong Ocean University (Zhanjiang, China) were selected as test materials, namely *Hemerocallis* ‘Golden Coffee Ruffles’ *Hemerocallis* ‘Prairie Belle’, *Hemerocallis* ‘Kimberly’ *Hemerocallis* ‘Water Dragon’ and *Hemerocallis fulva* (Figure 12). The first four cultivars are those that performed excellently in early introduction and cultivation, are highly resistant to adverse conditions, free from pests and diseases, and capable of normal flowering (Figure 12A–D). All were introduced by the Daylily Germplasm Resource Center of Shanghai University of Applied Science and Technology. The fifth cultivar was a widely used daylily cultivars in Zhanjiang (Figure 12E). From April to June 2024, pollen collection was conducted during the peak flowering period of each variety. Through preliminary observations, it was found that approximately 1–2 h after the flowers fully opened, the anthers gradually split open on both sides, releasing pollen. At this stage, the pollen viability was relatively high (this was confirmed in the subsequent 2.5 experiment). Therefore, pollen collection should be conducted within 1–4 h after the anthers naturally split open (pollen collection times vary by variety due to differing flowering periods; pollen collection for *H*. ‘Golden Coffee Ruffles’ is conducted in the evening). Use tweezers to gently shake the pollen into a 2-milliliter centrifuge tube and bring it back to the laboratory for testing.

### 4.2. Pollen Germination and Germination Medium

According to the preliminary preexperiment, compared with the solid medium, the liquid medium was more effective on daylily pollen isolated germination, so the liquid medium was selected as the medium for this experiment. The specific operation was as follows: The slide glass was placed in a culture dish and medium was added, pollen was sprinkled onto the culture medium with a cotton ball, and it was placed in a plant growth chamber at 25 °C for dark cultivation, and it was observed under the optical microscope with 4 times magnification (E5, Sunny Group, Shanghai, China), and the length of the pollen tube exceeding the diameter of pollen grains was taken. The germination results were counted after 4 h of incubation, and the observations were repeated three times (three slides) for a total of nine fields of view, with the number of pollen grains in each field of view not less than 50. The effects of different culture temperatures on pollen germination and pollen tube growth, place the culture dishes in plant growth chambers maintained at 14, 17, 20, 23, 26, 29, 32, 35, and 38 °C, respectively. After incubating for 30, 60, 90, 120, 180, and 240 min, remove the samples and measure the pollen germination rate. The length of pollen tubes was measured with a microscopic micrometer, and the length of 10 pollen tubes was measured on each slide, and the mean value and pollen tube elongation rate were calculated. Pollen germination rate = (number of pollen germinated grains/total number of pollen) × 100%.

### 4.3. Screening of Pollen Viability Determination Methods

Fresh pollen was collected at 8–10 a.m. Pollen viability was determined using three methods: 2.3.5-triphenyltetrazolium chloride (TTC), Iodine-potassium iodide (I_2_-KI) and Acetocarmine staining [8]. Live pollen cells produce dehydrogenase during respiration, which reacts with TTC solution to produce a red color; the darker the color, the stronger the vitality [47]. The I_2_-KI staining method assesses pollen vitality based on the staining of starch within the pollen, relying on the reaction between iodine and starch within the pollen to stain the pollen. The acetic acid carmine staining method assesses pollen viability based on the strength of various enzyme activities and the integrity of the cell membrane [48,49]. Combined with the results of the previous pollen in vitro germination experiments, the suitable staining methods were screened. The specific operation was as follows: Three kinds of dye were dropped on the slide in advance, and a small amount of pollen was taken with tweezers and mixed with the dye, Cover the slide and proceed with the following treatment according to the different staining methods (Table 5). Observe under an optical microscope (E5, Sunny Group, China). Pollen viability (%) = number of stained pollen grains/total number of observed pollen grains × 100%.

### 4.4. Effect of Different Collection Times and Storage Conditions on Pollen Viability

The pollen collection time was every 1 h from the first opening of the bud until the flowers withered. Pollen was collected with tweezers and placed in 2 mL centrifuge tubes for storage, three samples were collected at each time period and repeated three times, and their pollen viability was tested by TTC staining.

Pollen was collected at the optimal time and placed in 2 mL centrifuge tubes for storage. After the collected pollen was mixed well, it was sieved using a 150 μm metal filter to remove impurities. The sieved pollen had been divided into eight equal portions and divided into two treatments: dry and non-dry. Each treatment has four temperatures (25 °C, 4 °C, −20 °C, and −40 °C). The steps for drying and storing pollen were as follows: the sieved pollen was placed in a drying oven and dehydrated at 30 °C for 4 h, and after dehydration, it was wrapped in sulfuric acid paper and loaded into 5 mL centrifuge tubes, and then the centrifuge tubes were put into glass bottles filled with silica gel for sealing, and the pollen that was not subjected to the drying process was directly put into a refrigerator for storage after being sieved and loaded into the centrifuge tubes. After 0 d, 7 d, 15 d, 30 d, 45 d and 60 d of storage, pollen viability was determined by TTC staining, three repetitions were performed for each treatment (storage temperature). Before each experiment, pollen stored at −20 °C and −40 °C was thawed in a refrigerator at 4 °C for 6 h, and then transferred to a plant growth chamber at 25 °C for 2 h before TTC staining for viability [51].

### 4.5. Data Analysis

The data were initially organized by Microsoft Excel 2016 software. All data were statistically analyzed by using SPSS 22.0. The data from experiments were expressed as mean values ± standard deviation. The experimental design was based on orthogonal experiments, with a total of 9 treatments according to (L9(3^4^)) orthogonal table, set up with 4 factors: sucrose, H_3_BO_3_, KNO_3_, and Ca(NO_3_)_2_, with 3 levels for each factor (Table 6), means grouping was with Duncan’s test (*p* < 0.05). Data of different temperature, dyeing methodand storage time was subjected to one-way analysis (ANOVA), Differences between samples were determined by Least Significant Difference (LSD) and Duncan’s test (*p* < 0.05). The data on the effect of temperature on daylily pollen in vitro germination were analyzed by curve fitting, and the best-fit model was determined based on *p*-values and *R*^2^ values, and the effect of temperature was quantified. The minimum temperature (Tmin), optimum temperature (Topt), and maximum temperature (Tmax) for pollen germination and pollen tube growth were estimated the fitted equations [52]. Furthermore, linear regression and Pearson correlation coefficient analysis were used to quantify the relationship between the pollen viability estimates obtained from staining and in vitro pollen germination. Plotting was performed using GraphPad Prism 10.1.2 software.

## 5. Conclusions

The pollen germination rate and pollen tube growth of five different daylily cultivars in different culture media differed significantly, in which the most influential factor was the sucrose concentration. Different incubation temperatures had a significant effect on the pollen germination, pollen tube growth and elongation of daylily, and the optimal temperature for pollen germination was 24.08 °C, and the optimum temperature range for pollen tube growth was 24.07–25.66 °C. Compared with acetocarmine and I_2_-KI, TTC staining showed a high correlation with pollen germination rate, which can be used for rapid determination of daylily pollen viability, and the optimal time for daylily pollen collection was between the hours of 9:00–12: for the four cultivars of daylily, with the exception of *H*. ‘Golden Coffee Ruffles’, which was collected during the 9:00–12.00 a.m. time period. Daylily pollen was best preserved under ultra-low temperature storage conditions of −40 °C. Pollen that has undergone drying treatment has higher viability than pollen that has not undergone drying treatment. Among them, the storage effect of *H*. ‘Water Dragon’ pollen is significantly higher than that of other varieties, with pollen viability still at 41.29% after 60 days of dry storage. The results of the study provide a scientific basis for the selection of parents, pollen preservation and pollination timing optimization in daylily cross-breeding.

## Figures and Tables

**Table 1:** The analysis of the effect of different media on the pollen germination rate of 5 Daylily cultivars using anorthogonal assay test strategy (OATS).

No.	Source g·L^−1^	H_3_BO_3_ g·L^−1^	KNO_3_ g·L^−1^	Ca(NO_3_)_2_ g·L^−1^	Pollen Germination Rates of Different Daylily Cultivars/%				
					*H.* ‘Golden Coffee Ruffles’	*H.* ‘Prairie Belle’	*H.* ‘Kimberly’	*H.* ‘Water Dragon’	*Hemerocallis fulva*
1	50	0.1	0.06	0.2	36.43 ± 1.99 a	48.91 ± 3.08 a	42.78 ± 2.92 a	62.65 ± 3.34 a	28.82 ± 1.62 a
2	50	0.2	0.08	0.25	28.18 ± 3.32 b	30.75 ± 2.41 bc	33.36 ± 4.06 b	53.62 ± 5.46 b	16.55 ± 2.52 bc
3	50	0.3	0.1	0.3	18.72 ± 2.35 cd	35.01 ± 2.81 b	32.93 ± 2.95 b	39.24 ± 4.04 c	20.01 ± 1.81 b
4	100	0.1	0.08	0.3	24.34 ± 3.17 bc	25.03 ± 1.74 cd	27.13 ± 1.97 bc	32.12 ± 2.45 cd	14.45 ± 1.43 cd
5	100	0.2	0.1	0.2	25.63 ± 3.79 bc	16.4 ± 1.42 e	19.15 ± 1.58 cde	29.35 ± 3.22 de	17.73 ± 2.07 bc
6	100	0.3	0.06	0.25	19.67 ± 3.84 bcd	19.12 ± 1.65 de	15.01 ± 2.46 de	27.26 ± 2.15 de	20.55 ± 1.77 b
7	150	0.1	0.1	0.25	12.77 ± 2.2 de	33.22 ± 3.9 b	23.7 ± 3.37 cd	13.23 ± 1.43 g	10.23 ± 0.47 de
8	150	0.2	0.06	0.3	17.06 ± 2.08 cde	25.38 ± 2.83 cd	26.86 ± 4.12 bc	14.76 ± 1.69 fg	12.37 ± 2.13 cd
9	150	0.3	0.08	0.2	9.38 ± 1.81 e	22.78 ± 1.11 de	13.92 ± 2.51 e	22.51 ± 1.57 f	5.88 ± 1.07 e

Each column of data represents the mean ± standard deviation (SD) for different varieties on nine culture media. Different lowercase letters show significant differences at *p* = 0.05 level, whereas the same lower case letters indicate that the difference was not significant.

**Table 2:** Extreme variance analysis of the optimization test for pollen in vitro germination medium.

Cultivar	Factor Level	Source	H_3_BO_3_	KNO_3_	Ca(NO_3_)_2_
*H.* ‘Golden Coffee Ruffles’	K1	27.78 a	24.51 a	24.38 a	23.81 a
	K2	23.22 a	23.62 a	20.63 a	20.20 a
	K3	13.07 b	15.92 b	19.04 a	20.04 a
	*R*	14.71	8.59	5.35	3.77
*H.* ‘Prairie Belle’	K1	38.22 a	30.87 a	32.90 a	29.36 a
	K2	21.95 b	24.18 b	26.19 b	24.61 a
	K3	22.28 b	27.40 ab	23.36 b	28.48 a
	*R*	16.27	6.69	9.54	4.75
*H.* ‘Kimberly’	K1	36.36 a	31.20 a	28.22 a	25.28 a
	K2	20.43 b	26.46 ab	24.80 a	24.02 a
	K3	21.49 b	20.62 b	25.26 a	28.97 a
	*R*	15.93	10.58	3.41	4.95
*H.* ‘Water Dragon’	K1	51.84 a	36 a	34.89 a	38.17 a
	K2	29.58 b	32.58 a	36.08 a	31.37 a
	K3	16.83 c	29.67 b	27.27 b	28.70 a
	*R*	35.01	6.33	8.81	9.47
*Hemerocallis fulva*	K1	21.79 a	17.83 a	20.58 a	17.48 a
	K2	17.57 b	15.55 a	12.28 b	15.78 a
	K3	9.49 c	15.48 a	15.99 b	15.60 a
	*R*	12.30	2.35	8.29	1.87

Different lowercase letters indicate significant differences at the 0.05 level. Three levels of sucrose: K1 = 50 g/L, K2 = 100 g/L, K3 = 150 g/L; H_3_BO_3_: K1 = 0.1 g/L, K2 = 0.2 g/L, K3 = 0.3 g/L; KNO_3_: K1 = 0.06 g/L, K2 = 0.08 g/L, K3 = 0.1 g/L; Ca(NO_3_)_2_: K1 = 0.2 g/L, K2 = 0.25 g/L, K3 = 0.3 g/L. And *R* represents the difference in range between the maximum and minimum values.

**Table 3:** ANOVA results for the effects of different media on pollen germination of 5 daylily cultivars.

Variety	Factor	Variance of Square	df	Average of Square	F Value (F)	*p* Value
*H.* ‘Golden Coffee Ruffles’	Sucrose	3060.90	2	1530.45	21.25	0.000
	H_3_BO_3_	1204.59	2	602.29	8.36	0.001
	KNO_3_	406.98	2	203.49	2.83	0.066
	Ca(NO_3_)_2_	245.58	2	122.79	1.70	0.189
	Inaccuracies	5186.80	72	72.04		
*H.* ‘Prairie Belle’	Sucrose	4672.83	2	2336.42	59.03	0.000
	H_3_BO_3_	605	2	302.50	7.64	0.001
	KNO_3_	1296.89	2	645.44	16.38	0.000
	Ca(NO_3_)_2_	344.35	2	172.18	4.35	0.016
	Inaccuracies	2849.56	72	39.58		
*H.* ‘Kimberly’	Sucrose	4282.39	2	2141.19	26.49	0.000
	H_3_BO_3_	1517.64	2	758.82	9.39	0.000
	KNO_3_	185.26	2	92.63	1.15	0.324
	Ca(NO_3_)_2_	357.46	2	178.73	2.21	0.117
	Inaccuracies	5819.53	72	80.83		
*H.* ‘Water Dragon’	Sucrose	16,950.24	2	8475.12	98.99	0.000
	H_3_BO_3_	542.07	2	271.04	3.17	0.048
	KNO_3_	1234.32	2	617.16	7.21	0.001
	Ca(NO_3_)_2_	1286.40	2	643.20	7.51	0.001
	Inaccuracies	6163.81	72	85.61		
*Hemerocallis fulva*	Sucrose	2108.39	2	1054.20	29.38	0.000
	H_3_BO_3_	96.50	2	48.25	1.35	0.267
	KNO_3_	932.36	2	466.18	12.99	0.000
	Ca(NO_3_)_2_	57.92	2	28.96	0.81	0.450
	Inaccuracies	2583.49	72	35.88		

**Table 4:** Effect of different factor levels on the growth of pollen tubes.

Factor Level		Cultivar				
		*H.* ‘Golden Coffee Ruffles’	*H.* ‘Prairie Belle’	*H.* ‘Kimberly’	*H.* ‘Water Dragon’	*Hemerocallis fulva*
Sucrose	T1	1556.75 a	1665.25 a	1910 a	2674.25 a	1548.75 a
	T2	1220.5 b	1347.75 b	1599 b	1949.5 b	1007 b
	T3	887.25 c	1037.75 c	786 c	1179.5 c	536 c
	R	669.5	627.5	1124	1494.75	1012.75
H_3_BO_3_	T1	1300 a	1532.5 a	1562 a	2198.75 a	1244.75 a
	T2	1210.25 ab	1261.5 b	1395.5 ab	1871.75 b	1227.25 a
	T3	1154.25 b	1256.75 b	1337.5 b	1732.75 b	942 b
	R	145.75	275.75	224.5	466	302.75
KNO_3_	T1	1169.25 b	1491.25 a	1361 a	1996 a	1003.25 a
	T2	1179.75 b	1323.5 b	1340 a	1968.5 a	976.25 a
	T3	1315.5 a	1236 b	1594 b	1838.75 a	1112.25 a
	R	146.25	255.25	254	157.25	27
Ca(NO_3_)_2_	T1	1166.25 a	1326.75 a	1371.5 a	1875 a	1068.75 a
	T2	1235.75 a	1371 a	1469 a	2027 a	1017 a
	T3	1262.5 a	1353 a	1454.5 a	1901.25 a	1006 a
	R	96.25	44.25	97.5	152	62.75

Different lowercase letters indicate significant differences at the 0.05 level. Three levels of sucrose: T1 = 50 g/L, T2 = 100 g/L, T3 = 150 g/L; H_3_BO_3_: T1 = 0.1 g/L, T2 = 0.2 g/L, T3 = 0.3 g/L; KNO_3_: T1 = 0.06 g/L, T2 = 0.08 g/L, T3 = 0.1 g/L; Ca(NO_3_)_2_: T1 = 0.2 g/L, T2 = 0.25 g/L, T3 = 0.3 g/L. And R represents the range difference between the maximum and minimum values.

**Table 5:** Methods for pollen staining solutions and staining conditions.

Type of Dye Method	Post-Treatment	Judgment Basis of Active Pollen
0.5% TTC	30 min of dark culture at 30 °C [7]	red
0.5% I_2_-KI	Observe immediately [50]	red
1% Acetocarmine staining	Stand for 5 min [9]	red

**Table 6:** Determination of the composition of pollen germination medium for different daylily cultivars by orthogonal assay test strategy (OATS) = L_9_(3^4^).

Level	Factor			
	Sucrose/(g·L^−1^)	H_3_BO_3_/(g·L^−1^)	KNO_3_/(g·L^−1^)	Ca(NO_3_)_2_/(g·L^−1^)
1	50	0.1	0.06	0.2
2	100	0.2	0.08	0.25
3	150	0.3	0.1	0.3

## Data Availability

The raw data supporting the conclusions of this article will be made available by the authors on request.

## Supplementary Materials

The following supporting information can be downloaded at: https://www.mdpi.com/article/10.3390/plants14121854/s1, Table S1: Maximum germination rate, pollen tube length, and budgetary optimum, minimum, and maximum temperatures fitted to temperature response data; Table S2: Analysis of variance of effects of storage time, storage conditions and the interactions on pollen viability of 5 daylily cultivars.
