# Supplementary material for: Optimization of In Vitro Germination, Viability Tests and Storage of Daylily (Hemerocallis spp.) Pollen
Source: Plants (Basel). 2025 Jun 16;14(12):1854. doi: 10.3390/plants14121854 (PMC12197142; doi:10.3390/plants14121854)
Supplement: Supplementary file 1 [file plants-14-01854-s001.zip › plants-3659424-supplementary.pdf]

Supplementary Table S1. Maximum germination rate, pollen tube length, and budgetary optimum, minimum, and maximum temperatures fitted to temperature response data.

		Cultivars									
		H. 'Golden Coffee Ruffles'		*H.* 'Prairie Belle'		*H.* 'Kimberly'		*H.* 'Water Dragon'		*Hemerocallis fulva*	
Maximum germination rate/%		32.55		50.12		32.94		52.31		26.58	
Maximum pollen tube length/ μ m		1391.79		1720.60		1705.46		2506.41		1324.78	
temperature	Topt	25.14	25.53	26.67	25.33	24.08	24.43	25.45	25.72	25.76	25.05
	Tmin	12.69	13.78	11.21	11.24	10.91	11.34	11.78	12.42	13.26	13.50
	Tmax	37.59	37.28	42.12	39.41	37.25	37.52	39.11	39.02	38.26	36.59

Supplementary Table S2. Analysis of variance of effects of storage time, storage conditions and the interactions on pollen viability of 5 daylily cultivars.

Cultivar	**Factor**	**Variance of square**	**df**	**Average of square**	**F value (F)**	**P value**
H. 'Golden Coffee Ruffles'	Storage conditions	15671.32	7	2238.76	63.25	P<0.0001
	Storage time	116996.91	5	23399.38	661.083	P<0.0001
	Storage conditions×Storage time	11158.31	35	318.81	9	P<0.0001
	Inaccuracies	13591.87	384	35.40		
H. 'Prairie Belle'	Storage conditions	45005.26	7	6429.32	87.04	P<0.0001
	Storage time	149302.58	5	29860.52	404.24	P<0.0001
	Storage conditions×Storage time	20322.44	35	580.64	7.86	P<0.0001
	Inaccuracies	28365.64	384	73.87		
H. 'Kimberly'	Storage conditions	37834.79	7	5405.97	89.80	P<0.0001
	Storage time	155345.82	5	31069.16	516.17	P<0.0001
	Storage conditions×Storage time	14713.87	35	420.40	6.98	P<0.0001
	Inaccuracies	23113.60	384	60.19		
H. 'Water Dragon'	Storage conditions	72217.69	7	10316.81	140.39	P<0.0001
	Storage time	164834.73	5	32966.95	448.6	P<0.0001
	Storage conditions×Storage time	27490.88	35	785.45	10.69	P<0.0001

	Inaccuracies	28218.94	384	73.49		
Hemerocallis fulva	Storage conditions	15681.99	7	2240.28	64.85	P<0.0001
	Storage time	79809.82	5	15961.96	462.06	P<0.0001
	Storage conditions×Storage time	13248.78	35	378.54	10.96	P<0.0001
	Inaccuracies	13265.42	384	34.55		
